# Plasmid DNA Delivery Using a Stable Nanovesicle Platform: A Design-of-Experiments-Guided Investigation

**DOI:** 10.1021/acsbiomaterials.5c01328

**Published:** 2025-12-05

**Authors:** Mariana Köber, Irene González-Domínguez, Diego Valdospinos, Eduard Puente-Massaguer, Júlia Piqué-Ponti, David Piña, Laia Avilés-Domínguez, Ariadna Boloix, Miguel F. Segura, Nora Ventosa, Francesc Gòdia

**Affiliations:** † Institute of Materials Science of Barcelona (ICMAB-CSIC), 16719 Universitat Autònoma de Barcelona, Cerdanyola del Vallès, 08193 Barcelona, Spain; ‡ Centro de Investigación Biomédica en Red in the Subject Area of Bioengineering, Biomaterials and Nanomedicine (CIBER-BBN), 28029 Madrid, Spain; § Departament d’Enginyeria Química Biològica i Ambiental, Universitat Autònoma de Barcelona, Cerdanyola del Vallès, 08193 Barcelona, Spain; ∥ Childhood Cancer and Blood Disorders Group, Vall d’Hebron Institut de Recerca (VHIR), Universitat Autònoma de Barcelona, 08035 Barcelona, Spain; ∇ Nanomol Technologies S.L., Campus UAB, Bellaterra 08193, Spain

**Keywords:** plasmid DNA delivery, nonviral vectors, lipid-based nanovesicles, quatsomes, HEK293 cell transfection, Design of Experiments, green fluorescent protein

## Abstract

Delivering plasmid DNA (pDNA) into cells is essential for numerous biotechnological and biomedical applications. Among available nanocarriers, nonviral lipid-based vesicles are particularly promising for transfecting mammalian cells. Nevertheless, further development is required to create delivery systems that are both broadly effective across cell types and scalable for clinical use. Here, we explore stable nanovesicles composed of the sterol derivative cholesteryl *N*-(2-dimethylaminoethyl)­carbamate (DC–CHOL) and myristalkonium chloride (MKC) as a platform for pDNA delivery. These nanovesicles, previously shown to efficiently deliver small RNAs to neuroblastoma cells, exhibit favorable physicochemical properties, such as high morphological uniformity and long-term colloidal stability, positioning them as strong candidates for DNA transfection. Using suspension-adapted human embryonic kidney 293 (HEK293) cells, which are widely employed for producing viral vectors and complex biotherapeutics, we evaluated the delivery performance of DC–CHOL/MKC nanovesicles with a reporter plasmid encoding enhanced green fluorescent protein. A Design of Experiments (DoE) approach was applied to identify and optimize critical transfection parameters, namely, the DNA concentration, DNA-to-vesicle ratio, and NaCl concentration in the complexing medium. This study demonstrates the capability of these nonviral vectors to deliver double-stranded plasmid DNA and emphasizes the critical role of the physicochemical characteristics of the pDNA/lipid complex in achieving efficient transfection.

## Graphical abstract

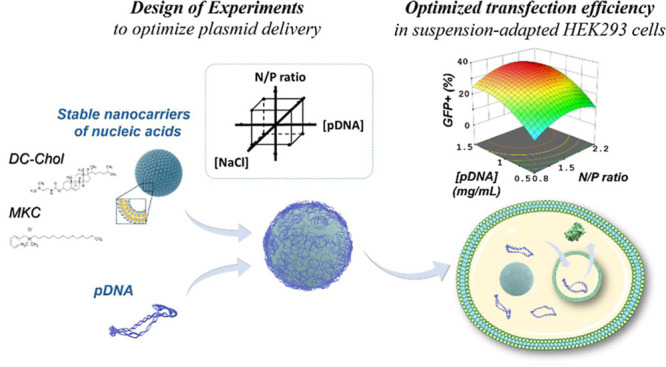

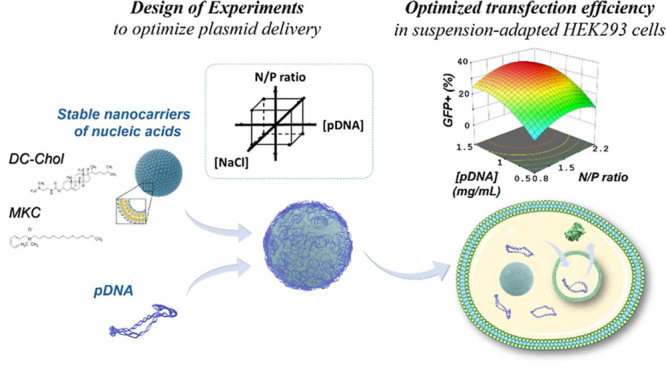

## 1 Introduction

Nonviral vectors have demonstrated significant potential for delivering nucleic acids in both biotechnological and biomedical contexts.^1,2^ Among them, lipid-based nanocarriers have emerged as a leading technology,^3,4^ as evidenced by the clinical approval of lipid nanoparticles (LNPs) for mRNA and siRNA delivery in treatments for COVID-19 ^5,6^ and hereditary transthyretin amyloidosis.^7^ These systems are based on nanoparticles with controllable size and shape that can load nucleic acids and deliver them into the cytoplasm or nucleus of the cell. An easy control of molecular composition, low immunogenic effects, and a simple manufacturing pipeline^8^ are some of the main advantages of these delivery systems compared to traditional viral vectors.

Among the different nonviral candidates, quatsome nanovesicles (QS) are a promising candidate for the delivery of nucleic acids. QS are organic lipid-based nonliposomal nanovesicles composed of sterols and quaternary ammonium surfactants, conferring a positive surface charge to these nanovesicles. Different QS formulations showing a remarkable morphological homogeneity and colloidal stability over years^9^ have been developed in the past decade,^10–12^ and their potential for different diagnostic and therapeutic applications has been demonstrated in several works,^13–15^ including the delivery of nucleic acids.^12^ For the latter, a protonable tertiary amine is added to the formulation through the sterol derivative cholesteryl *N*-(2-dimethylaminoethyl)­carbamate (DC-cholesterol, p*K*_a_ ≈ 7.8), which has shown to promote the efficient delivery of small RNAs (sRNAs) to different cancer cells, e.g., microRNAs into human neuroblastoma cell cultures^12^ and siRNAs into ovarian cancer cell cultures,^16^ leading to the desired changes in the target protein levels. DC-QS are protonated at physiological pH and interact with the negative charges of sRNAs, leading to complex formation and concomitant protection of the sRNA from nuclease degradation.

In this work, we have explored the use of pH-sensitive QS for the delivery of double-stranded plasmid DNA (pDNA) to produce proteins of interest in human cell cultures (Figure 1). In contrast to the delivery of sRNAs, plasmid DNA needs to reach the cell nucleus to be transcribed to RNA and then translated to protein. For this proof of concept, we have studied transient gene expression in the HEK293 cell line, which is one of the most widely used cell platforms for protein production, including small peptides, monoclonal antibodies, and viral vectors,^17^ since HEK293 cells grow fast, can be cultured in serum-free media, and are capable of maintaining human post-translational modifications.^18^

**Figure 1:**
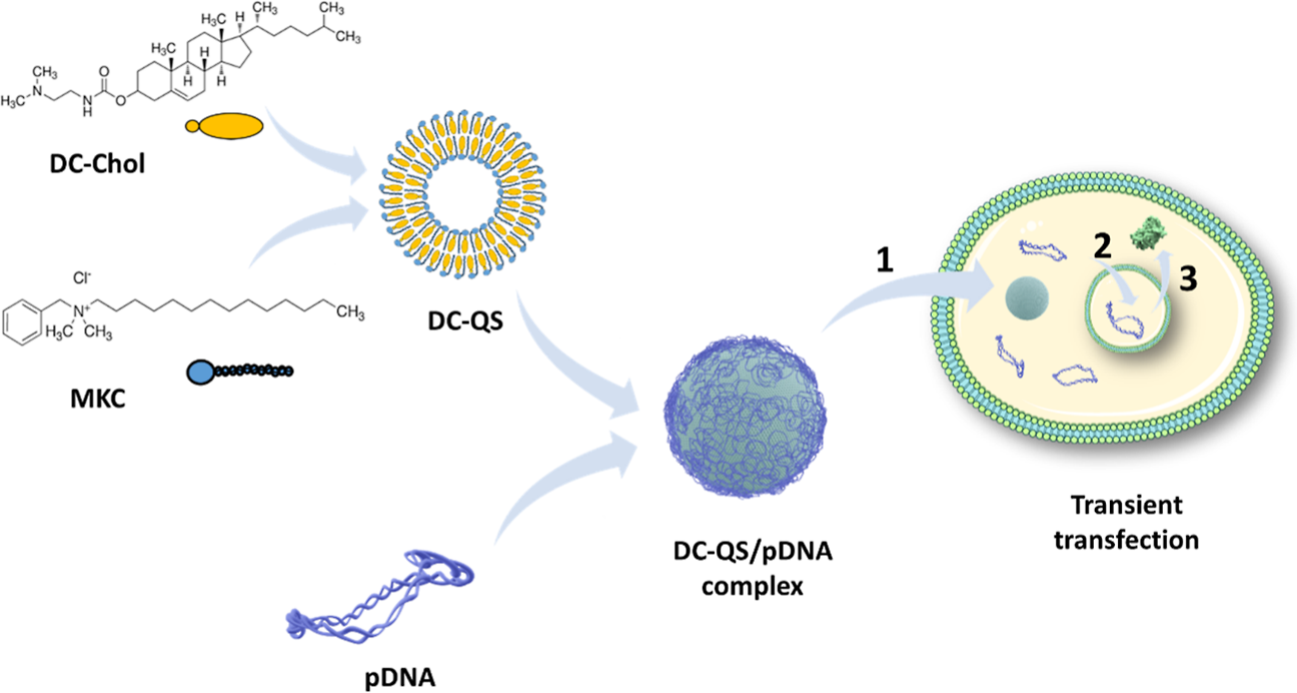
Scheme illustrating the composition of DC-QS, the complexation of pDNA on the DC-QS surface, and transfection into eukaryotic cells, followed by the main steps required to achieve protein expression: crossing of the plasma membrane of the pDNA/DC-QS complex (1), entry of pDNA into the nucleus (2), pDNA transcription, and recombinant protein expression (3).

As known from other nonviral vector transfection reagents, the physicochemical attributes of the pDNA/DC-QS complexes play a fundamental role in the delivery process, and variables like the mixing ratio of pDNA and transfection agent, the pDNA dose, and the details of the preparation protocol need to be optimized.^19^ While the prevailing standard is to encapsulate nucleic acids within the particle, it has been shown that the complexation of RNA on the exterior of ionizable cationic lipid nanoparticles can yield equivalent performance in vitro and in vivo.^20^

Transfection efficiency generally depends on different and possibly interdependent experimental variables. To optimize the transfection efficiency for optimal protein production, we have employed a Design of Experiments (DoE) strategy. In contrast to optimizations performed changing one variable at a time, the DoE strategy facilitates a rational exploration of the multivariable parameter space within an appropriate statistical framework, reducing the number of experiments that need to be carried out to identify the best condition.^21–24^

To facilitate recombinant protein quantification, we used a pDNA encoding the enhanced green fluorescent protein (GFP) as a reporter protein, for which the easily measurable fluorescence intensity can be converted into a GFP concentration. By doing so, we were able to develop an optimized protocol for the delivery of pDNA containing 6257 bp into HEK293 cells, which could be used for the production of recombinant proteins for future clinical use.

## 2 Materials and Methods

### 2.1 Cloning of the Gene of Interest and pDNA Preparation

A double-stranded circular pDNA encoding the intracellular enhanced GFP was used as a reporter. The pOPINE-eGFP pDNA was generated in the Protein Expression Core Facility of the Institute for Research in Biomedicine (IRB) in Barcelona.^25,26^ The pDNA was produced and purified as previously described.^27^

### 2.2 DC-QS Preparation

The DC-QS used here were developed by A. Boloix et al.^12^ DC-QS were prepared using the DELOS-SUSP (Depressurization of an Expanded Liquid Organic Solution-Suspension) technology.^28–30^ DC-QS were prepared by adding a solution of 0.065 M cholesteryl *N*-(2-dimethylaminoethyl)­carbamate (DC-Chol) in ethanol to a high-pressure vessel, which is then pressurized upon the introduction of CO_2_, creating an expanded organic solution. Afterward, the CO_2_-expanded solution is depressurized over the aqueous phase, consisting of 0.008 M of benzyldimethyltetradecylammonium chloride (MKC) in Milli-Q H_2_O. One week after vesicle production, the DC-QS were purified by tangential flow filtration using the KrosFlo Research Iii TFF System (Spectrum Laboratories, Repligen Corporation; Waltham, Massachusetts, USA) using diafiltration columns “C02-E-100” with a membrane pore size of 100 kDa (Repligen Corporation; Waltham, Massachusetts, USA), removing the remaining ethanol and free surfactant.^12^

### 2.3 DC-QS/pDNA Complex Formation

The DC-QS/pDNA complexes were formed by diluting DC-QS to the desired concentration in PBS solution (1 × 0.0067 M PO_4_, HyClone. GE Healthcare, Logan, UT, USA) while vortexing for 10 s. Plasmid DNA was subsequently added to the DC-QS solution, followed by three 1 s vortexing cycles to ensure thorough mixing.

### 2.4 N/P Ratio Estimation

The number of phosphate groups in the double-stranded plasmid DNA was estimated based on its base pairs (bp) count (6257 bp). A concentration of 100 μg/mL of DNA (molecular weight (MW) = 38,629 kDa) corresponds to a concentration of 1.56 × 10^13^ plasmids/mL and 1.96 × 10^17^ P groups/mL.

The number of nitrogen groups in DC-QS was calculated from the number of MKC molecules present in the mother solution. The MW of the MKC is 368.04 g/mL and the final concentration of MKC was estimated to be 1270 μg/mL, taking into account the overall recovery of membrane components of 43% (determined by gravimetric analysis) and assuming an equimolar ratio of MKC and DC-Chol in the final product. Consequently, the overall MKC concentration corresponds to 2.08 × 10^18^ molecules/mL, of which about half of the molecules, 1.04 × 10^18^ N groups/mL, were assumed to expose their ammonium groups on the outer vesicle surface, accessible for nucleic acid complexation.

For the transfection reagent PEI, the nitrogen/phosphate (N/P) ratio was calculated as the theoretical amount of amine groups that are mixed with the phosphate groups in the plasmid DNA in a 1:2 (w/w) ratio (Bono et al., 2020).^19^ The number of amine groups in the 25 kDa linear PEI (PolySciences, Warrington, PA, USA) was calculated assuming an average number *n* of monomers in the polymer chain, where each monomer contains one amino group, as shown in its chemical structure provided by the manufacturer. With an MW of the monomer of 43 g/mol, *n* = 581. Thus, a concentration of 20 μg/mL corresponds to concentrations of 4.8 × 10^14^ PEI polymers/mL and 2.8 × 10^17^ N groups/mL.

### 2.5 Particle Size and ζ-Potential Measurements

Particle size and ζ-potential of the different DC-QS/DNA and PEI/DNA complexes were determined using the dynamic light scattering and electrophoretic light scattering techniques, respectively, and a Zetasizer Nano ZS (Malvern Instruments, Malvern, United Kingdom), employing a He–Ne 633 nm laser and detecting backscattered light at 173°. The hydrodynamic diameter was derived at 298 K and 0.8872 cP, using disposable plastic cuvettes (Scharlab S.L., Barcelona, Spain) and a sample volume of 1 mL. ζ-Potential measurements were performed at 298 K, using DTS1070 disposable folded capillary cuvettes. Measurements were carried out in triplicate, and reported values refer to the average ± SD.

### 2.6 Cryogenic Transmission Electron Microscopy (Cryo-TEM) Imaging

Cryo-TEM images were acquired with a JEOL JEM 2011 transmission electron microscope (JEOL, Tokyo, Japan) at 200 kV. Samples were placed on a holey carbon grid, vitrified by plunge-freezing in liquid ethane, and stored in liquid nitrogen until loading onto a cryogenic sample holder (Gatan 626 CTH, Gatan, USA). The working temperature was kept below 98 K. Images were recorded using a Gatan UltraScan US1000 CCD camera and analyzed with Digital Micrograph software.

### 2.7 TNS Assay

The p*K*_a_ of DC-QS was determined by using the TNS assay. The TNS molecule (2-(*p*-toluidino)-6-naphthalene sulfonic acid) is a negatively charged fluorophore whose fluorescence is quenched in water but whose emission increases upon its partition into positively charged lipid membranes via electrostatic interactions. Here, the fluorescence intensity was measured across a pH range of 4.0–10.0 and fitted to a Boltzmann sigmoidal function. Three buffer solutions were prepared to cover the whole pH range, using 10 mM sodium acetate (pH 4.0–5.5), Bis-Tris (pH 6.0–7.5), or Tris (pH 8.0–10.0), adjusted with HCl or NaOH and supplemented with 150 mM NaCl. A 1200 μM stock solution of TNS (Merck Life Science S.L.U., Spain) was prepared in DMSO and diluted into each buffer to reach a final concentration of 6 μM. In black 96-well plates (Sarstedt S.A.U., La Roca del Vallès, Spain), DC-QS were diluted into the buffer-TNS mixtures to achieve a final concentration of 75 μM DC-Chol (the ionizable lipid) in a total volume of 200 μL. The fluorescence intensity was measured using a plate reader (Infinite M Nano+, Tecan Trading AG, Switzerland) at an excitation wavelength of 325 nm, an emission wavelength of 435 nm, and a working range of 400–460 nm, with the temperature set to 37 °C. The apparent p*K*_a_ was determined as the pH corresponding to half-maximal fluorescence intensity.

### 2.8 Cell Line and Culture Conditions

The cell line used is a serum-free suspension adapted HEK293SF-3F6 (NRC, Montreal, Canada), provided by Dr. Amine Kamen (McGill University, Montreal, Canada). The cells were cultured in HyClone HyCell TransFx-H culture medium (GE Healthcare, Chicago, IL, USA), supplemented with 0.1% (v/v) Pluronic F-68 (Gibco, Life Technologies, Thermo Fisher, San José, CA, USA) and 4 mM Glutamax (Gibco). The cells were maintained at a 20 mL final volume in an exponential growth phase in 125 mL disposable polycarbonate Erlenmeyer flasks (Corning, New York, USA) shaken at 130 rpm in a humidified incubator at 37 °C and 5% CO_2_ in air. Cell concentration and viability were measured using a NucleoCounterNC-3000 (Chemometec, Allerod, Denmark) cytometer, following the protocol established by the manufacturer.

### 2.9 Transient Transfection

HEK293 cells were grown until 2 × 10^6^ cells/mL and were transiently transfected using DC-QS as a DNA delivery vector. The final concentration of DNA was changed according to each specific experiment. DC-QS/DNA complexes that had been incubated for 15 min at RT were added to the cell culture. Transfection with 25 kDa linear polyethylenimine (PEI) was used as positive control, using a DNA/PEI 1:2 (w/w) ratio corresponding to an N/P ratio of 14, as previously described.^31^ Experiments were performed in 6-well plates in duplicate, and responses were analyzed 48 h post transfection (hpt). For the first assessment of the transfection efficiency in HEK293 cells, different N/P ratios and different concentrations of DC-QS or pDNA were analyzed. The experiments consisted in testing different N/P ratios from 0.2 to 7, changing the concentration of DNA in the complex formation and maintaining the concentration of DC-QS constant and vice versa, as presented in Table S1. Different responses were analyzed, such as cell viability, cell number, the percentage of transfected cells, and the overall GFP concentration.

### 2.10 Flow Cytometry

A sample of 300 μL was taken and centrifuged at 300*g* for 5 min, and the supernatant was removed. Cells were then fixed by adding 300 μL of formaldehyde 2% to form a homogeneous mixture. All the samples were incubated for 10 min at room temperature and then centrifuged at 500*g* for 5 min in which the supernatant was removed. Finally, 300 μL of PBS (phosphate-buffered saline) 1× solution was added to the pellet for its subsequent analysis by flow cytometry. The percentage of GFP-positive was measured with a flow cytometer BD FACS Canto (BD Biosciences, San Jose, CA, USA) with a two-laser configuration at 488 and 635 nm. The laser emitting at 488 nm was used for GFP measurements, and fluorescence was detected with a GFP FITC-A detector. A total of 20.000 events were analyzed for each experimental condition. The results were analyzed with the software FACS DIVA (BD Biosciences).^32^

### 2.11 GFP Quantification

Samples of 500 μL were taken from the cell culture and centrifuged at 1000*g* for 5 min. The supernatant was separated from the pellet, and both were stored at −20 °C until further analysis. The cell pellet was subjected to three freeze/thaw cycles (2 h frozen at −20 °C and thawed at 37 °C for 30 min). The pellet was vortexed for 5 s between cycles. Lysed pellets were resuspended in 0.5 mL of TMS buffer (50 mM Tris–HCL, 150 mM NaCl, 2 mM MgCl_2_, pH 8.0) and centrifuged at 13,700*g* for 20 min^33^ The GFP concentration was analyzed by fluorimetry using a fluorescence spectrophotometer (Agilent Technologies, Santa Clara, CA, USA) at room temperature at an excitation wavelength of λ_exc_ = 488 nm and registering the fluorescence at the emission wavelength λ_em_ = 510 nm. Relative fluorescence units (RFU) were calculated by subtracting the measured fluorescence unit values of the culture medium of HEK293 cells without transfection. RFU values obtained on different days were normalized using a 0.1 mg/mL quinine sulfate solution as internal control. The final GFP concentration was calculated with the following expression, obtained from a previously performed calibration:^33^

GFP(mg/L)=(RFU−1.864)/61.905

### 2.12 Full Factorial Design

To define the workspace of the main variables, we conducted a 2^4^ full factorial DoE with a value range based on preliminary studies^34^ and literature. Central points (0,0,0,0) were included to account for internal regions of the workspace that could have levels superior to those of the design space limit values (−1,–1,–1,–1) and (1,1,1,1) (see Table S3 for further details). A pseudoquadratic term (curvature) was included for this purpose. Responses under analysis were cell growth, cell viability, and cell transfection efficiency. The selected independent variables were DC-QS/pDNA incubation time, pDNA concentration, NaCl concentration, and N/P ratio. Data were analyzed with R software. Experiments were performed in duplicate.

### 2.13 Central Composite Design and Desirability Functions

To determine the optimal value for each screened variable, a five-level three-factor central composite design (CCD) was used.^35^ The ranges defined for each selected variable were based on the 2^4^ full factorial DoE results. Two different responses were analyzed: cell transfection efficiency and GFP production. Data obtained for each response was fitted to a second-order polynomial equation by linear regression analysis with Design-Expert 11 software. An overall optimum from the considered responses was obtained by the combination of the individual optima using desirability functions. In short, relevance values (*s*-value) of 5, 3, 4, and 5 were given to the viability, cell concentration, transfection efficiency, and protein production, respectively, with the goal to maximize cell transfection efficiency and GFP production models, and an iteration process was conducted to determine the best conditions integrating the different responses of the study.^23^

### 2.14 Statistical Analysis

The statistical analysis of the different models generated was performed by using Design-Expert 11 software. The quality of the regression of the fitted equations was evaluated with the *R* ^2^ and adjusted *R* ^2^ coefficients. An analysis of variance (ANOVA) *F* test was used to determine the significance of the equations, and the individual coefficients were assessed with a *t*-test. The lack-of-fit test was used to evaluate differences between experimental measurements and values predicted by the model and pure error (variation among replicates) of the fitted equations. In all analyses, *p* values of 0.05 were considered statistically significant.

## 3 Results

### 3.1 Physicochemical Characterization of pDNA/DC-QS Complexes

pH-sensitive DC-QS nanovesicles for nucleic acid delivery were prepared using the scalable and environmentally friendly DELOS-SUSP technology.^12,28,29^ DC-QS are composed of the sterol derivative cholesteryl *N*-(2-dimethylaminoethyl)­carbamate (DC-Chol), a weak base with a tertiary amine, and myristalkonium chloride (MKC), an European Medicines Agency (EMA)-approved derivative of the quaternary ammonium surfactant benzalkonium chloride (BAK, EMA/CHMP/495 737/2013), which at an equimolar ratio form unilamellar nanovesicles in pure water. These nanovesicles are pH-sensitive, with a p*K*_a_ of approximately 7.5, and display high size and morphological homogeneity with an average diameter of ∼50 nm (Figure S1). Due to the permanent positive charges of the quaternary amines of MKC and the protonated tertiary amines of DC-Chol, these nanovesicles interact electrostatically with negatively charged nucleic acids and form DC-QS/dsDNA complexes. As previously shown for sRNA, the tertiary amine is a key component mediating intracellular release.^12^

To study DNA transfection, we used a double-stranded circular pDNA of 6257 bp that codes for the enhanced GFP. First, we assessed conjugate size and ζ-potential at different ratios of DC-QS and pDNA (specified in Table S1), as the physicochemical DC-QS/pDNA complex properties directly impact transfection efficiency.^32,35,36^ The different ratios of DC-QS and pDNA are expressed as the nitrogen/phosphate (N/P) ratio, for which we only considered the quaternary ammonium groups from the MKC in the DC-QS formulation as they yield a pH-independent charge contribution. Depending on pH, the tertiary ammonium groups from DC–CHOL may be protonated, yielding an additional positive charge.

As expected, conjugate sizes showed a clear dependence on the N/P ratio, remaining below 200 nm at both high and low ratios (Figure 2A,B and Table S2) as a result of electrostatic repulsion between equally charged particles (Figure 2C and Table S2) while increasing to the micrometer range at N/P ratios close to 1, where the reduced net surface charge is insufficient to sustain such repulsion and clusters comprising multiple DC-QS/pDNA complexes are formed. Importantly, size dispersion, despite showing a slight increase toward N/P ratios close to 1, always remained low, with PDI values not exceeding 0.35 (Figures 2C and S2). Cryo-TEM images of DC-QS/pDNA complexes at an N/P ratio of 1.5 show the presence of pDNA on the outer vesicle surface (Figure 2D) and suggest the possible presence of some vesicle clusters. Taken together, the physicochemical characterization indicates a stable nanovesicle–plasmid DNA complex formation in the time window that is relevant for an extemporaneous preparation (before use).

**Figure 2:**
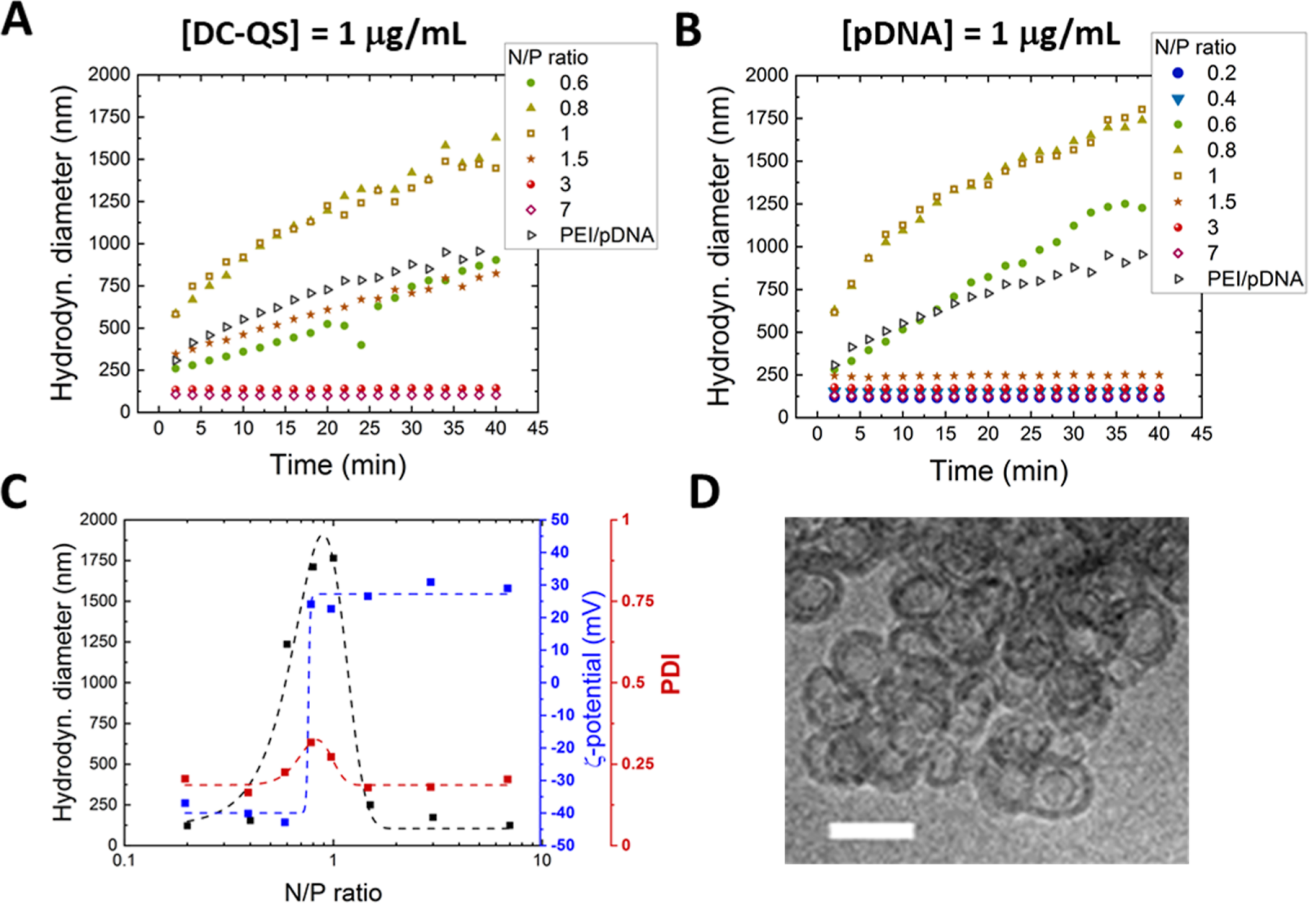
Physicochemical characterization of DC-QS/pDNA conjugates in PBS buffer containing 100 mM NaCl. (A,B) Hydrodynamic diameter of DC-QS/pDNA conjugates during 40 min of incubation in PBS at different N/P ratios, at constant DC-QS concentration and constant pDNA concentration, respectively. (C) Hydrodynamic diameter, PDI, and ζ-potential of DC-QS/pDNA conjugates after 40 min of incubation at a constant pDNA concentration of 1 μg/mL. Lines are drawn to guide the eye and do not represent any type of model fit. (D) Cryo-TEM image of DC-QS/pDNA complexes at an N/P ratio of 1.5 in PBS after 15 min of incubation. The scale bar represents 100 nm.

### 3.2 First Screening of Cell Viability and Transfection Efficiency

Various DC-QS/pDNA conjugates were evaluated for their ability to deliver pDNA in vitro. An internal positive control using 25 kDa linear polyethylenimine (PEI) as a transfection reagent was added for comparison, which was optimized in a previous work.^31,37^ Following that optimization strategy, first screenings were performed preparing DC-QS/pDNA complexes in PBS containing 100 mM NaCl and incubating these for 15 min prior to their addition to the cell culture. Transfected cells were then incubated for 48 h and assessed for cell growth and viability, as well as GFP expression by flow cytometry. In this screening, we tested a range of N/P ratios (from 0.2 to 7), both at a constant pDNA concentration of 1 μg/mL and at a constant DC-QS concentration of 1 μg/mL, respectively (Figure 3 and Table S3). For a constant pDNA concentration of 1 μg/mL and N/P ratios in the range of 0.2–1.5 (corresponding to DC-QS concentrations of 5–36 μg/mL), cell viability remained above 93% (Figure 3A), while higher N/P ratios yielded a strong decline in cell viability due to increased DC-QS concentrations (>∼70 μg/mL). Interestingly, cell viability in this case was 5 times higher than that reported for DC-QS/miRNA complexes in a neuroblastoma cell line (Ic_50_ ≈ 14 μg/mL),^12^ possibly related to the different nature of plasmid DNA vs miRNA and different cell origin and cell culture mode (adherent vs suspension cell cultures). On the other hand, overall GFP expression peaked at N/P ratios of 1.5 and 3, with the highest mean fluorescence intensity (MFI) observed at these conditions (Figure 3B). Among these two conditions, an N/P ratio of 1.5 seems the most appropriate for this pDNA concentration considering the decrease in cell viability at an N/P ratio of 3 (Figure 3A). For the constant DC-QS concentration of 1 μg/mL, cell viability was high for all N/P ratios (Figure 3C), but the transfection efficiency was relatively low, likely due to the lower pDNA and DC-QS concentrations used (Figure 3D). Of note, despite the lower percentage of GFP-expressing cells obtained with DC-QS compared to PEI, the stronger fluorescence intensity per cell indicates that DC-QS promotes a more effective intracellular release and expression of the delivered plasmid. Collectively, these results demonstrate that DC-QS nanovesicles can deliver pDNA into the cell nucleus under conditions that maintain cell viability. On the other hand, the N/P ratio and overall pDNA concentration have a large impact on transfection efficiency. To perform a more systematic optimization of the transfection efficiency, we performed multivariable optimization using a DoE.

**Figure 3:**
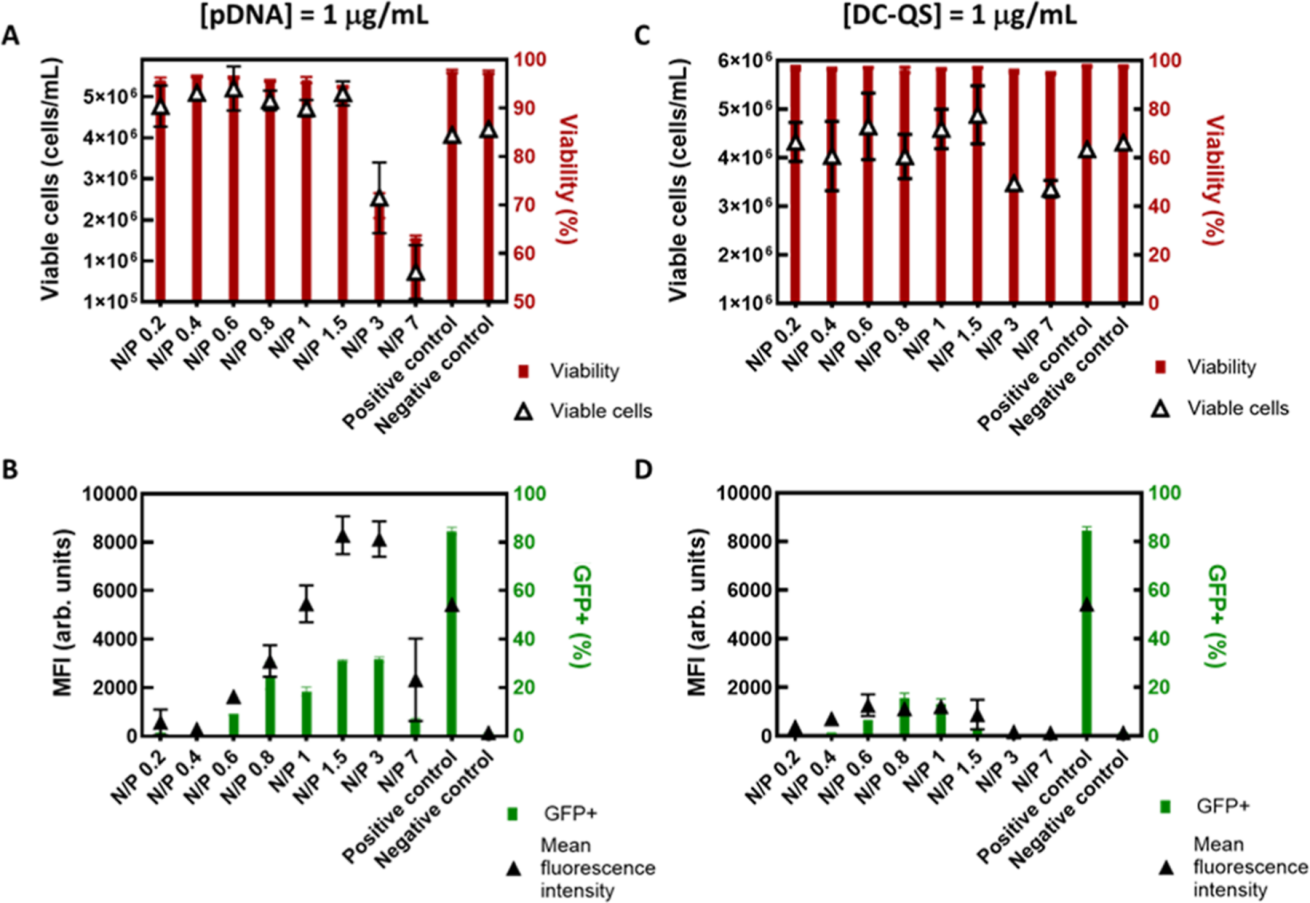
In vitro experiments to determine the workspace for pDNA transfection using DC-QS. Impact of the N/P ratio on cell viability and transfection efficiency in HEK293 cells. A constant pDNA concentration of 1 μg/mL (A,B) and a constant DC-QS concentration of 1 μg/mL were used (C,D). (A,C) Viable cell concentration and fraction of viable cells, and (B,D) percentage of GFP-positive cells and GFP production indicated by the MFI of the positive population. A positive control consisting of transfecting cells with the transfection reagent PEI and a negative control (nontransfected cells) were added for comparison. Experiments were performed in duplicate, and mean values ± standard deviation are shown. DC-QS/pDNA complexation was performed in PBS solution containing 100 mM NaCl and incubated for 15 min.

### 3.3 Optimization of DNA Delivery by DC-QS Using Design of Experiments

Among the different variables involved in our study, we kept the culture media composition and culture conditions constant (Figure 4A) while varying the parameters that were observed to affect the transfection efficiency and the physicochemical properties of the DC-QS/pDNA conjugates. These include the N/P ratio, the overall pDNA concentration, and the incubation time. As the NaCl concentration in the complexing media can also impact the complex formation between pDNA and DC-QS, the NaCl concentration was included as a fourth variable (Figure 4A). To assess the impact of these variables, we evaluated four different responses: cell viability, cell growth, percentage of GFP-positive cells, and the overall GFP protein production. A two-step DoE was employed, with an initial screening step to identify the variables that have a significant impact on the output parameters and determine the workspace for subsequent optimization. The aim of the second step is to determine the variable values that yield optimal cell transfection (Figure 4B). Finally, a validation experiment was carried out to corroborate the predicted maximal level of GFP expression.

**Figure 4:**
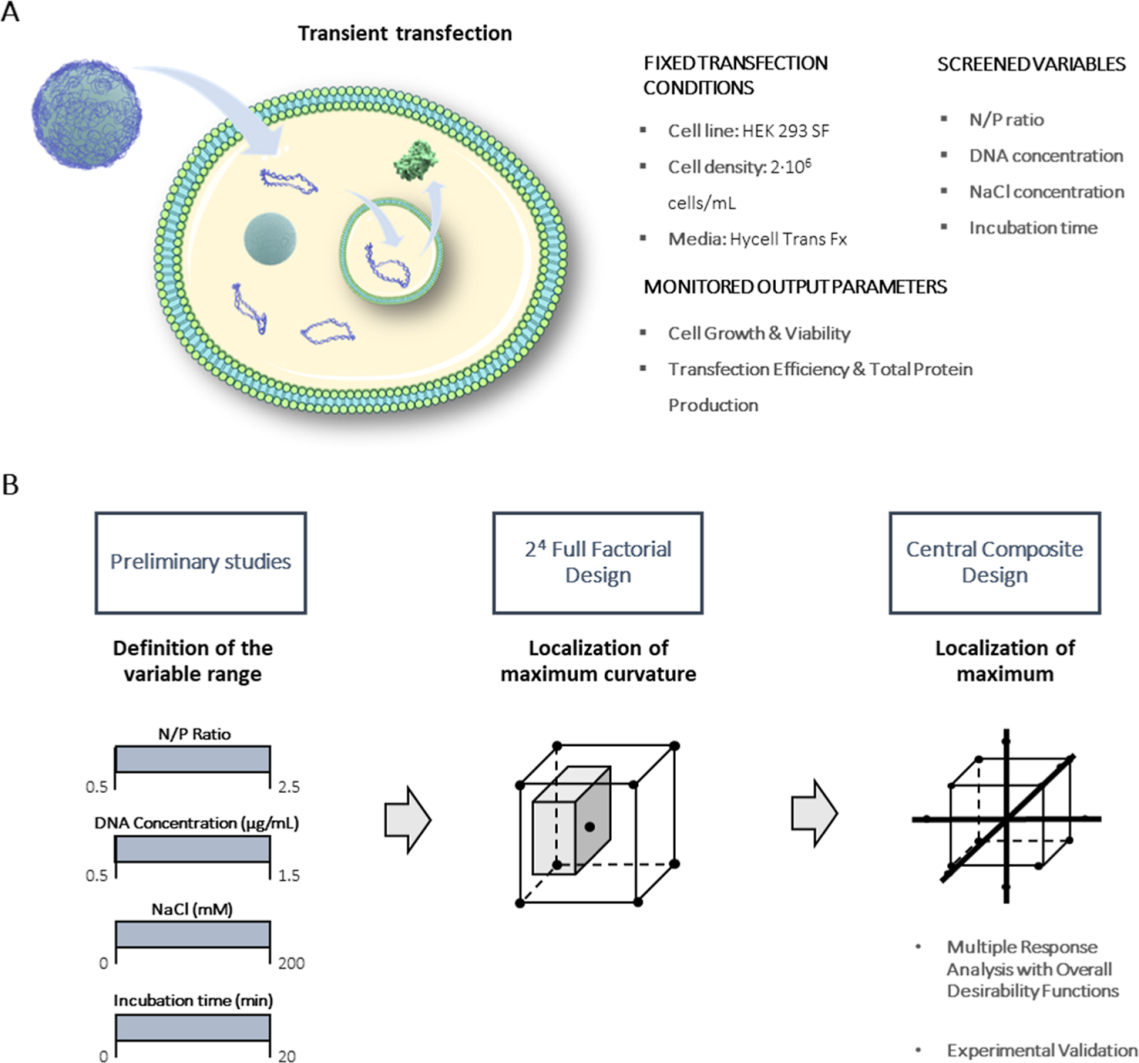
(A) Experimental design, screened variables, and monitored output parameters. (B) DoE workflow applied for a systematic optimization of transfection in HEK cells using DC-quatsomes.

#### 3.3.1 Screening of Design Space Using a Full Factorial Design

To identify the variables with the greatest impact on cell transfection efficiency, a 2^4^ full factorial design was employed (Figure 4B). The design included central points to qualitatively assess potential nonlinearity in the response. The screened variables in this first round were (i) pDNA concentration, (ii) N/P ratio, (iii) NaCl concentration, and (iv) incubation time. The ranges for pDNA concentration, N/P ratio, and incubation time (Table 1) were selected based on the preliminary studies of this work (Figures 2 and 3), while the value range of NaCl concentration was based on literature.^35^ Table S4 lists the 18 combinations of variable values tested (2^4^ = 16 combinations, plus 2 central points), as well as the measured responses cell viability, cell growth, transfection efficiency (fraction of GFP + cells) (Figure S4A–C).

**Table 1:** Selected Variable Ranges for the 2^4^ Full Factorial Design to Study the Cell Transfection Efficiency of HEK293 Cells

screened variable	min. value	central point	max. value
[pDNA] (μg/mL)	0.5	1	1.5
N/P ratio	0.5	1.5	2.5
[NaCl] (mM)	0	100	200
incubation time (min)	1	10	20

Three different response models were generated using Design-Expert 11 software (Table S2). The statistical significance of each model was confirmed using ANOVA, and a refinement process was applied to eliminate statistically nonsignificant terms according to the hierarchy principle.^38^ The models indicate that the transfection efficiency was mainly affected by the N/P ratio, the pDNA concentration, and the NaCl concentration (Table S4 and Figure S4D), while the DC-QS/pDNA incubation time only impacted cell growth. Overall, the best transfection conditions were found at the central point (0, 0, 0), yielding 17 ± 2% of GFP + expressing cells (Table S4 and Figure S4C).

#### 3.3.2 Optimization

To determine the variable values for a maximal transfection efficiency more precisely, we used a CCD with three variables, consisting of a 2^3^ full factorial design augmented with 6-star points (Figure 4B). Based on the initial screening results, which showed maximum transfection at the central point and minimal influence of incubation time, the workspace for the different parameters impacting cell transfection efficiency was defined using a fixed incubation time of 10 min and narrowing the ranges for pDNA concentration, N/P ratio, and NaCl concentration (Table 2). GFP production was included as a second response variable. The obtained data were fitted to second-order polynomial models by linear regression analysis (Figure 5 and Table S5).

**Table 2:** Experimental Matrix for the Three-Factor CCD^a^

screened variable	level 1	level 2	level 3	level 4	level 5
N/P ratio	0.3	0.8	1.5	2.2	2.7
[pDNA] (μg/mL)	0.2	0.5	1	1.5	1.8
[NaCl] (mM)	16	50	100	150	184

a The five levels correspond to codified values 0, ±1, and ±1.68.

**Figure 5:**
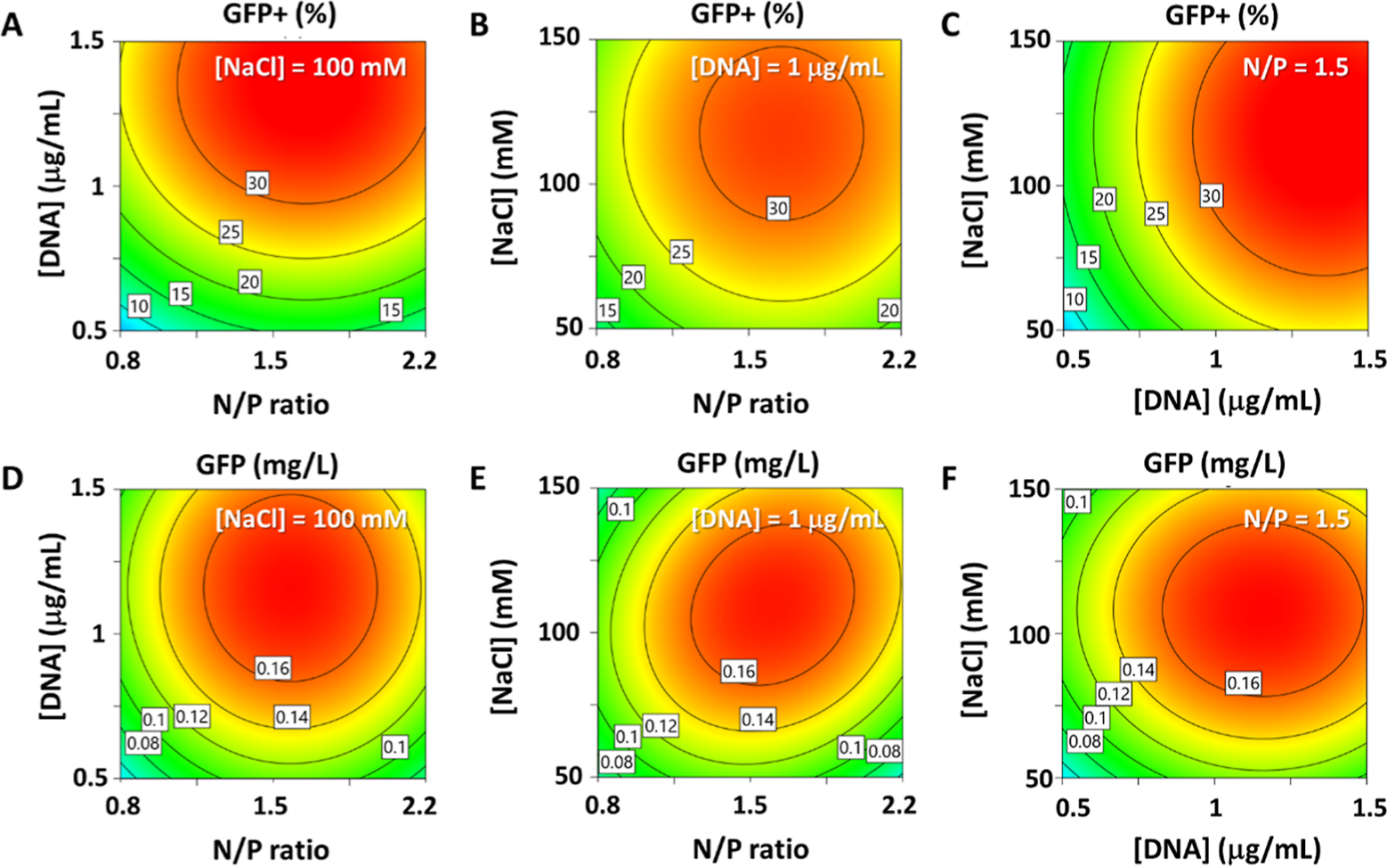
Response contour plots of cell transfection efficiency and GFP expression in HEK293 cells 48 hpt, as a function of pDNA concentration, N/P ratio, and NaCl concentration. (A–C) Fraction of cells expressing detectable amounts of GFP. (D–F) Detected GFP concentration.

Figure 5 shows the response surface for cell transfection efficiency and GFP production 48 hpt as a function of pDNA concentration, N/P ratio, and NaCl concentration. All three variables influence the transient transfection of HEK293 cells 48 hpt, with the optimal GFP production around the central point variable combination in the CCD. Moreover, a correlation between the N/P ratio and the NaCl concentration was observed (Figure 5E). Table S5 summarizes all of the statistical parameters of the CCD for each response. The viability was mainly impacted by the NaCl concentration (Table S5, Model A), whereas the cell growth was affected by both the NaCl concentration and DNA concentration (Table S5, Model B). On the other hand, the percentage of transfected cells and the absolute protein production were affected by the three variables (Table S5, Model C and Model D).

A combined optimal condition was determined using a desirability functions approach with the aim to maximize both cell transfection efficiency and GFP production.^23^ The optimal condition was predicted to be a pDNA concentration of 1.2 μg/mL, an N/P ratio of 1.6, and a NaCl concentration of 109 mM (Table 3). Using these conditions, experimental responses for transfection efficiency and GFP yield agreed with the response predicted by combining both models, which were 34 ± 7% and 0.18 ± 0.01 mg/L, respectively (Table 3). At the optimal condition, the DC-QS/pDNA complex exhibited an average size of 250 ± 3 nm, a polydispersity index (PDI) of 0.223 ± 0.013, and a ζ-potential of 13 ± 2 mV.

**Table 3:** Optimal GFP Expression Predicted by the CCD for an N/P Ratio of 1.6, a pDNA Concentration of 1.2 μg/mL, and a NaCl Concentration of 109 mM^a^

response	transfection efficiency (%)	GFP production (mg/L)
model prediction	34 ± 7	0.18 ± 0.01
experimentally observed	28 ± 1	0.163 ± 0.003

a The experimentally obtained values are also included.

## 4 Discussion

Nonviral vectors for nucleic acid delivery have been extensively studied due to their potential in biomedical and biotechnological applications. Quatsomes present several advantages in this context, including high morphological homogeneity and exceptional colloidal stability under storage and physiological conditions, which ensure consistent performance over time. Notably, their scalable and reproducible production via compressed fluid technologies such as DELOS-SUSP makes them suitable for both research and industrial applications. The DC-quatsome formulation in particular has previously been shown to efficiently deliver sRNAs (miRNAs and siRNAs) to different cancer cell lines (human neuroblastoma^12^ and ovarian cancer cell cultures^16^). Here, we expand the applicability of DC-quatsomes as a nanocarrier platform for the delivery of nucleic acids, positioning quatsomes as a robust and versatile platform for plasmid DNA delivery in mammalian cell systems as well. It is important to mention that myristalkonium chloride (MKC) is a European Medicines Agency (EMA)-approved derivative of the quaternary ammonium surfactant benzalkonium chloride (BAK, EMA/CHMP/495 737/2013), and DC–CHOL has been used in several phase I trials (e.g., NCT00009841). Furthermore, previous work demonstrated that intravenous administration of quatsomes composed of CHOL/MKC in mice produced no detectable histological changes in organs with major quatsome uptake.^11^

Compared to the delivery of sRNAs previously reported for this nanovesicle platform, plasmid DNA must reach the cell nucleus to be transcribed into RNA and then translated to protein. For an efficacious pDNA delivery, several barriers must be overcome: (i) internalization of the pDNA/lipid complex into the cells, (ii) cytosolic delivery of the pDNA, and (iii) its nucleocytoplasmic transport. These processes are influenced by multiple factors, particularly the physicochemical properties of the pDNA/lipid complex and the specific cell line used.^19^ In this work, we focused on identifying variables that influence the physicochemical characteristics of the pDNA/lipid complex and found that the pDNA concentration, N/P ratio, and NaCl concentration in the complexing medium are critical parameters for efficient transfection with DC-QS nanovesicles.

Optimal transfection was achieved with DC-QS/pDNA complexes with an average diameter of ∼250 nm (PDI ∼0.2) and a moderately positive ζ-potential of ∼13 mV. These results are in line with previous studies reporting improved cellular uptake of nanoparticles with moderate positive ζ-potentials due to favorable interactions with the negatively charged cell membrane. However, increasing amounts of the cationic reagent per cell can lead to cytotoxicity.^39,40^ In this regard, both the N/P ratio and NaCl concentration significantly influence complex size and ζ-potential, thereby modulating interactions with the cell membrane and affecting internalization pathways.^41^ The key role of NaCl has also been demonstrated in DNA/PEI systems, where ions facilitate an increase in the transfection efficiency.^3^ For DC-QS, we identified an optimal NaCl concentration of ∼110 mM using the DoE approach.

Beyond cellular uptake, both cytosolic delivery and/or nucleocytoplasmic transport of pDNA appear to be strongly influenced by the pDNA concentration, N/P ratio, and NaCl concentration, as evidenced by the dependence of GFP yield per cell on these variables. It is well established that the N/P ratio affects endosomal escape,^42^ which is likely also true for DC-QS-mediated transfection. Following successful release into the cytoplasm, pDNA must overcome diffusional and metabolic barriers to reach the nucleus, with a substantial fraction of intact pDNA often degraded before reaching the nuclear envelope (NE).^43^ Moreover, pDNA needs to cross the NE to reach the nucleus, which is likely facilitated during mitosis when the NE breaks down, leading to more effective DNA transcription in proliferating cells compared to nonproliferating cells.^44^

Additional variables not addressed in this study could also impact transfection efficiency,^18,45^ such as specific pDNA characteristics or moieties that interact with proteins on or in the cell, potentially influencing take, trafficking, and nuclear import.

In summary, we demonstrate that nanovesicles composed of DC–CHOL and MKC are capable of delivering not only sRNAs to neuroblastoma and ovarian cancer cells^12,16^ but also pDNA to suspension-adapted HEK293 cells. For targeted nucleic acid delivery to specific cells and tissues, a systematic optimization of the formulation would be required,^46^ as the nanocarrier composition profoundly affects the nucleic acid encapsulation efficiency, stability, uptake, endosomal escape, and release. The DoE approach is a valuable tool for this purpose, as it enables efficient screening with a reduced number of experiments, which is particularly relevant when animal testing is involved.^47^

## 5 Conclusions

We demonstrated that nanovesicles composed of MKC and DC–CHOL can efficiently deliver plasmid DNA for recombinant protein production in suspension-adapted mammalian cells. A DoE approach enabled systematic screening of different conditions for cell transfection and GFP production in a reliable and time-efficient manner. The critical variables influencing transfection yields were the pDNA concentration, the ratio of pDNA and DC-QS, and the NaCl concentration in the complexation medium, while transfection yields did not show any statistically significant dependence on the incubation time. Optimal transfection efficiency and GFP expression were achieved using 1.2 μg/mL pDNA, an N/P ratio of 1.6, and ∼110 mM NaCl in the complexation media. On the other hand, pDNA concentrations below 1 μg/mL and nanovesicle concentrations above 36 μg/mL proved suboptimal, due to either reduced transfection efficiency or increased cytotoxicity, respectively. These findings support the potential of DC-QS nanovesicles as an effective delivery system for large nucleic acids and underscore the importance of further optimization in HEK293 and other cell lines.

## Supplementary Material



## Data Availability

The data supporting this article have been included as part of the Supporting Information.

## Notes

Additional experimental details, including physicochemical characterization of DC-QS nanovesicles; size dispersion of DC-QS/pDNA conjugates; kinetics of cell viability after transfection with DC-QS/pDNA; cell viability, cell growth and transfection efficiency obtained in the first DOE optimization round (24 full factorial design); concentrations of pDNA and DC-QS used in the initial screening; data shown in Figure 2A–C and Figure 3A–D; ANOVA statistical analysis of full factorial design; and experimental matrix, results, and ANOVA analysis of the CCD (PDF)

## ⊥

Department of Microbiology, Icahn School of Medicine at Mount Sinai, New York, New York, 10029, USA
