# Supplementary material for: Plasmid DNA Delivery Using a Stable Nanovesicle Platform: A Design-of-Experiments-Guided Investigation
Source: ACS Biomater Sci Eng. 2025 Dec 5;12(1):354–64. doi: 10.1021/acsbiomaterials.5c01328 (PMC12801183; doi:10.1021/acsbiomaterials.5c01328)
Supplement: Supplementary file 1 [file ab5c01328_si_001.pdf]

# Supplementary Information

## Plasmid DNA Delivery using a Stable Nanovesicle Platform: A Design-of-Experiments-Guided Investigation

**Mariana Köber[1,2,*,†], Irene González-Domínguez[3,*,x,†] Diego Valdospinos[1,3], Eduard Puente-Massaguer[3,x], Júlia Piqué-Ponti[1], David Piña[1,2], Laia Avilés-Domínguez[1], Ariadna Boloix[4], Miguel F. Segura[4], Nora Ventosa[1,2], Francesc Gòdia[3]**

[1]Institute of Materials Science of Barcelona (ICMAB-CSIC), Universitat Autònoma de Barcelona, Cerdanyola del Vallès, 08193, Barcelona, Spain

[2]Centro de Investigación Biomédica in the subject area of Bioengineering, Biomaterials and Nanomedicine (CIBER-BBN), 28029, Madrid, Spain

[3]Departament d'Enginyeria Química Biològica i Ambiental, Universitat Autònoma de Barcelona, Cerdanyola del Vallès, 08193, Barcelona, Spain

[4]Childhood Cancer and Blood Disorders Group, Vall d'Hebron Institut de Recerca (VHIR), Universitat Autònoma de Barcelona, Barcelona, Spain

[†]Contributed equally to this work.

[x] Current address: Department of Microbiology, Icahn School of Medicine at Mount Sinai, New York, New York, 10029, USA

[*] mkober@icmab.es; irene.gonzalez@mssm.edu

**This file includes:**


| | | |
|---|---|---|
| Figure S1 | Physico-chemical characterization of DC-QS nanovesicles | Page 2 |
| Figure S2 | Size dispersion of DC-QS/pDNA conjugates | Page 3 |
| Figure S3 | Kinetics of cell viability after transfection with DC-QS/pDNA | Page 4 |
| Figure S4 | Cell viability, cell growth and transfection efficiency obtained in the first DOE optimization round (24 full factorial design) | Page 5 |
| Table S1 | Concentrations of pDNA and DC-QS used in the initial screening | Page 6 |
| Table S2 | Data shown in Figure 2A, 2B and 2C | Page 7 |
| Table S3 | Data shown in Figure 3A-3D | Page 9 |
| Table S4 | Full factorial design, responses and ANOVA statistical analysis | Page 11 |
| Table S5 | Experimental matrix, results and ANOVA analysis of the equations obtained in the CCD | Page 14 |

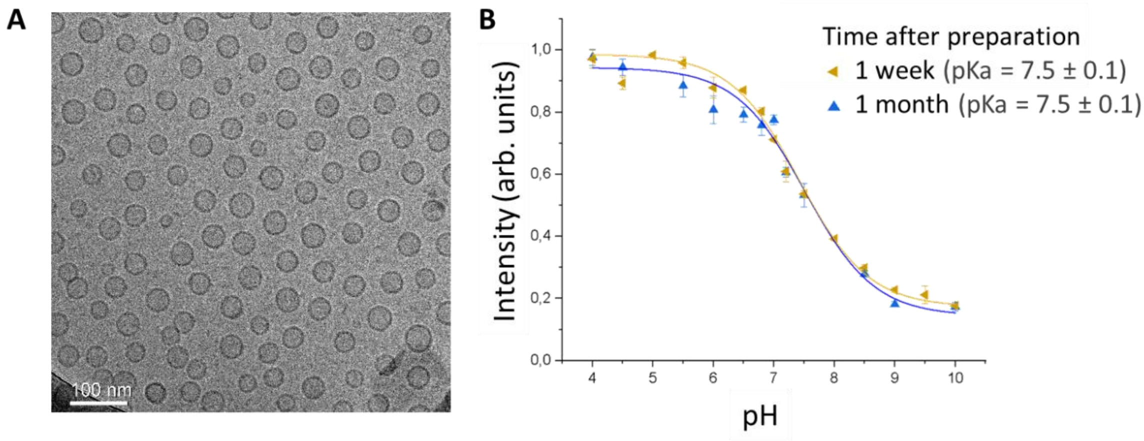


**Figure S1:** Physico-chemical characterization of DC-QS nanovesicles. A) cryo-TEM image of DC-QS showing highly homogeneous unilamellar morphology and size of the nanovesicles. B) pH dependence of the fluorescence emission of 6-(p-toluidino)-2-naphthalenesulfonic acid sodium salt (TNS), yielding the pKa of DC-QS nanovesicles.

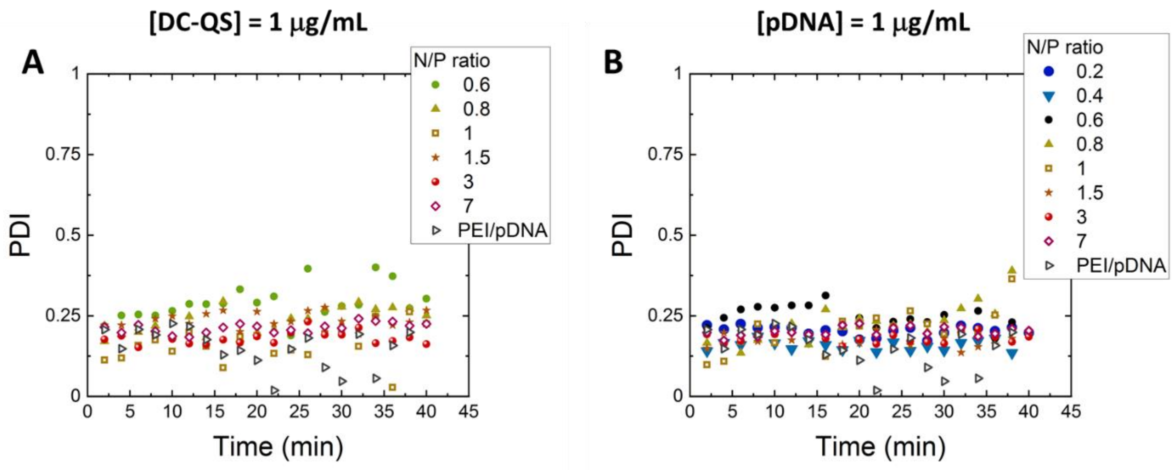


**Figure S2**: Temporal evolution of size dispersion of DC-QS/pDNA conjugates after incubation in PBS buffer containing 100 mM NaCl, expressed as PDI measured by Dynamic Light Scattering. (A, B) PDI of DC-QS/pDNA conjugates during 40 min of incubation in PBS at different N/P ratios, at constant DC-QS concentration, and constant pDNA concentration, respectively.

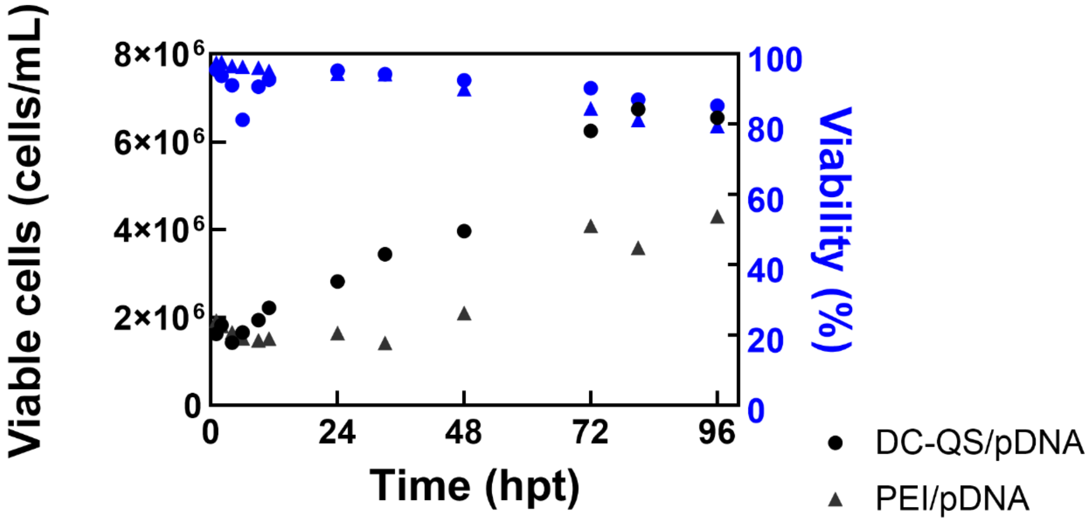


**Figure S3:** Kinetics of cell viability after transient transfection of HEK293 cells with DC-QS/pDNA at an N/P ratio of 1 and with PEI/pDNA.

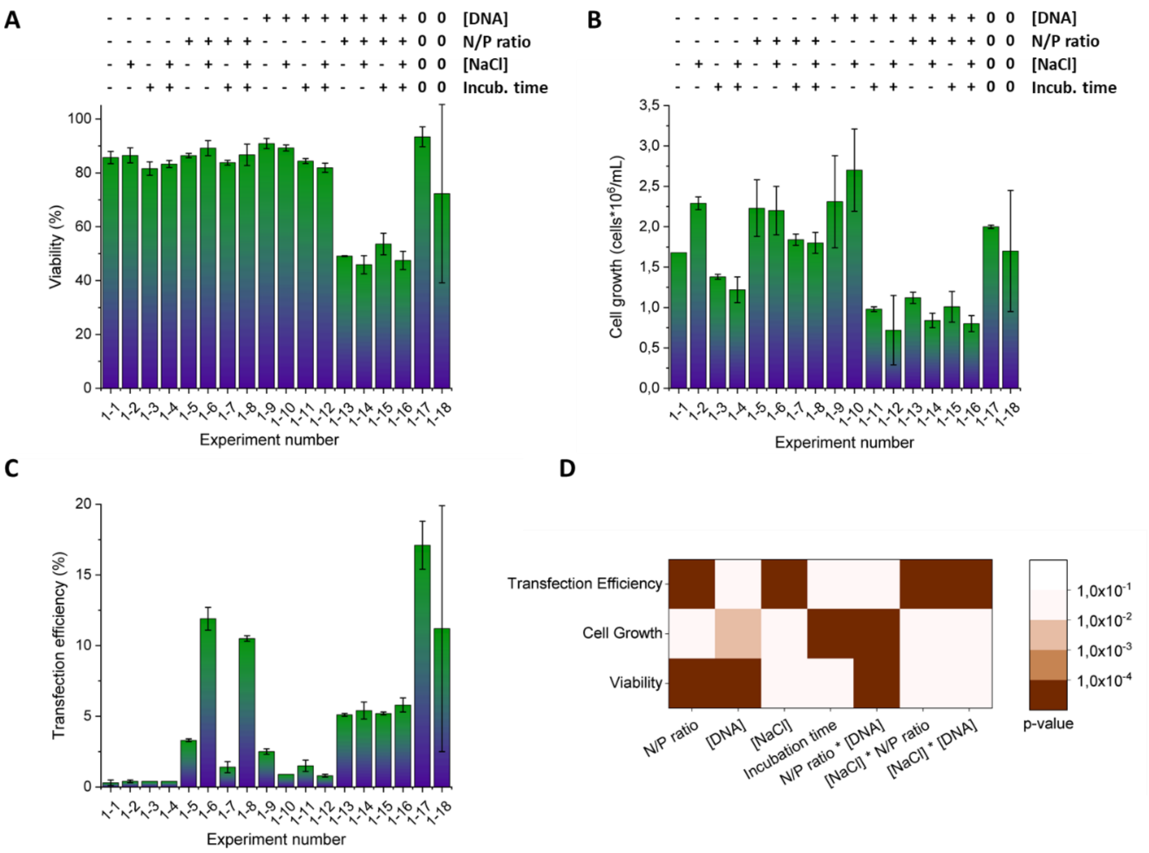


**Figure S4: (A-C)** Cell viability, cell growth and transfection efficiency obtained for the 18 combinations of variable values tested in the first optimization round ($2^4$ full factorial design). Uncertainty is expressed as the standard deviation obtained from replicates. For better comprehension, on top we have indicated the parameter value combination for each experiment number ("-" refers to the minimal value, "0" to the central point, and "+" to the maximal value indicated in **Table 1**). (D) Heat map of correlations (*p*-value) from ANOVA. Variables with a *p*-value > 0.05 are excluded from the ANOVA model.

**Table S1:** Concentrations of pDNA and DC-QS used in the initial screening covering N/P ratios ranging from 0.2 to 7.

| **1 µg/mL of pDNA in the culture** | | | |
|---|---|---|---|
| **N/P ratio** | **Phosphate groups per mL** | **Quaternary amines per mL** | **[DC-QS] (µg/mL)** |
| 0.2 | $1.96 \cdot 10^{16}$ | $3.91 \cdot 10^{15}$ | 4.8 |
| 0.4 | $1.96 \cdot 10^{16}$ | $7.82 \cdot 10^{15}$ | 9.6 |
| 0.6 | $1.96 \cdot 10^{16}$ | $1.17 \cdot 10^{16}$ | 14.4 |
| 0.8 | $1.96 \cdot 10^{16}$ | $1.56 \cdot 10^{16}$ | 19.1 |
| 1.0 | $1.96 \cdot 10^{16}$ | $1.96 \cdot 10^{16}$ | 23.9 |
| 1.5 | $1.96 \cdot 10^{16}$ | $2.93 \cdot 10^{16}$ | 35.9 |
| 3.0 | $1.96 \cdot 10^{16}$ | $5.87 \cdot 10^{16}$ | 71.7 |
| 7.0 | $1.96 \cdot 10^{16}$ | $1.37 \cdot 10^{17}$ | 167.4 |
| **1 µg/mL of DC-QS in the culture** | | | |
| **N/P ratio** | **Phosphate groups per mL** | **Quaternary amines per mL** | **[pDNA] (µg/mL)** |
| 0.2 | $8.18 \cdot 10^{15}$ | $4.09 \cdot 10^{16}$ | 4.2 |
| 0.4 | $8.18 \cdot 10^{15}$ | $2.05 \cdot 10^{16}$ | 2.1 |
| 0.6 | $8.18 \cdot 10^{15}$ | $1.36 \cdot 10^{16}$ | 1.4 |
| 0.8 | $8.18 \cdot 10^{15}$ | $1.02 \cdot 10^{16}$ | 1.0 |
| 1.0 | $8.18 \cdot 10^{15}$ | $8.18 \cdot 10^{15}$ | 0.84 |
| 1.5 | $8.18 \cdot 10^{15}$ | $5.45 \cdot 10^{15}$ | 0.56 |
| 3.0 | $8.18 \cdot 10^{15}$ | $2.73 \cdot 10^{15}$ | 0.28 |
| 7.0 | $8.18 \cdot 10^{15}$ | $1.17 \cdot 10^{15}$ | 0.12 |

**Table S2:** Data shown in Figure 2A, 2B and 2C, determined using Dynamic Light Scattering. Hydrodynamic diameter of DC-QS/pDNA conjugates monitored during 40 min of incubation in PBS at different N/P ratios, at constant DC-QS concentration (Figure 2A), and constant pDNA concentration (Figure 2B). Figure 2C: Hydrodynamic diameter and ζ-potential of DC-QS/pDNA conjugates after 40 min of incubation at a constant pDNA concentration of 1 μg/mL.

| Data shown in **Figure 2A** | | | | | | | |
|---|---|---|---|---|---|---|---|
| **Time** (min) | **z-average** (nm) | | | | | | |
| | **N/P = 0.6** | **N/P = 0.8** | **N/P = 1** | **N/P = 1.5** | **N/P = 3** | **N/P = 7** | **PEI 100 mM** |
| 2 | 259.5 | 586.8 | 580.9 | 345.6 | 134.8 | 106.7 | 307.7 |
| 4 | 279.1 | 668.6 | 747.1 | 375.1 | 136.4 | 102.6 | 413 |
| 6 | 307.4 | 749.3 | 805.9 | 411.6 | 139.3 | 103.4 | 457.6 |
| 8 | 331.8 | 810.5 | 890.9 | 428.2 | 135.8 | 99.61 | 506.5 |
| 10 | 359.1 | 909.8 | 918.8 | 461.3 | 138.5 | 98.55 | 551.9 |
| 12 | 382.9 | 984.6 | 1004 | 495.6 | 138.5 | 100 | 591 |
| 14 | 415.9 | 1047 | 1064 | 518.3 | 135.6 | 100.9 | 620.4 |
| 16 | 442.9 | 1104 | 1086 | 552.7 | 137.4 | 99.04 | 667.5 |
| 18 | 470.9 | 1136 | 1129 | 578.9 | 136.6 | 98.95 | 706.2 |
| 20 | 524.2 | 1195 | 1224 | 608.6 | 141.2 | 99.24 | 727.4 |
| 22 | 513.1 | 1282 | 1170 | 624.7 | 141.7 | 98.53 | 780.7 |
| 24 | 399.8 | 1322 | 1241 | 670.3 | 140.6 | 99.44 | 783.5 |
| 26 | 629.3 | 1321 | 1314 | 674.3 | 140.8 | 101.1 | 798.8 |
| 28 | 679.2 | 1319 | 1248 | 727.8 | 142.4 | 101.5 | 834.9 |
| 30 | 745.1 | 1421 | 1329 | 707.4 | 143.4 | 101.8 | 877.8 |
| 32 | 781.3 | 1383 | 1377 | 730 | 144 | 102 | 850.1 |
| 34 | 781.8 | 1581 | 1487 | 795.3 | 142.2 | 102.5 | 949.8 |
| 36 | 838.5 | 1475 | 1452 | 745 | 141.7 | 103.4 | 906.2 |
| 38 | 868.5 | 1506 | 1469 | 800.2 | 141.2 | 103 | 954.5 |
| 40 | 903.5 | 1628 | 1447 | 823.7 | 143 | 103.6 | |

| Data shown in **Figure 2B** | | | | | | | | | |
|---|---|---|---|---|---|---|---|---|---|
| **Time** (min) | **z-average** (nm) | | | | | | | | |
| | **N/P = 0.2** | **N/P = 0.4** | **N/P = 0.6** | **N/P = 0.8** | **N/P = 1** | **N/P = 1.5** | **N/P = 3** | **N/P = 7** | **PEI 100 mM** |
| 2 | 121.9 | 153.7 | 281.3 | 629.5 | 615.2 | 245.2 | 176.9 | 129.6 | 307.7 |
| 4 | 119.4 | 150.7 | 331.5 | 769.4 | 783.3 | 239.8 | 173.3 | 126.2 | 413 |
| 6 | 118.7 | 150.8 | 394.2 | 931.3 | 931.6 | 235.7 | 171.8 | 124.1 | 457.6 |
| 8 | 117.9 | 148.4 | 445.1 | 1026 | 1071 | 241 | 173.4 | 123.3 | 506.5 |
| 10 | 117.1 | 149 | 516.1 | 1095 | 1125 | 240.6 | 170.1 | 123.4 | 551.9 |
| 12 | 116.8 | 149.2 | 570.5 | 1157 | 1216 | 245 | 171.7 | 124 | 591 |
| 14 | 117 | 148.4 | 632.2 | 1258 | 1292 | 242 | 170.7 | 121.8 | 620.4 |
| 16 | 116.6 | 150.3 | 708.6 | 1330 | 1336 | 244.7 | 171.3 | 122.9 | 667.5 |
| 18 | 117.9 | 150.7 | 791.3 | 1354 | 1371 | 248.3 | 171.6 | 123.5 | 706.2 |
| 20 | 117.6 | 149.4 | 822.5 | 1406 | 1361 | 250.5 | 171.7 | 123.5 | 727.4 |
| 22 | 118.5 | 150.6 | 888.6 | 1464 | 1441 | 248.5 | 172.4 | 124.2 | 780.7 |
| 24 | 120.7 | 150.4 | 902.8 | 1521 | 1485 | 243.8 | 171 | 124.1 | 783.5 |
| 26 | 118.7 | 152 | 981.4 | 1554 | 1510 | 247 | 171.4 | 123.2 | 798.8 |
| 28 | 118.7 | 153.3 | 1026 | 1559 | 1531 | 250.9 | 172 | 123.5 | 834.9 |
| 30 | 119.1 | 154.3 | 1122 | 1617 | 1566 | 252 | 171.8 | 122.8 | 877.8 |
| 32 | 120.1 | 152 | 1199 | 1650 | 1607 | 248 | 171.6 | 124.2 | 850.1 |
| 34 | 120.4 | 153.1 | 1232 | 1697 | 1741 | 247 | 174.1 | 125.6 | 949.8 |
| 36 | 121.4 | 153.5 | 1250 | 1697 | 1753 | 251.6 | 172.9 | 123.8 | 906.2 |
| 38 | 121.7 | 156.4 | 1227 | 1740 | 1802 | 249.5 | 174.4 | 124.9 | 954.5 |
| 40 | 121.1 | | | | | 250.3 | 171.6 | 123.9 | |

| Data shown in **Figure 2C** | | | | |
|---|---|---|---|---|
| **N/P ratio** | **z-average** (nm) | | **ζ-potential** (mV) | |
| 0.2 | 121.4 | 0,3 | -37 | 8 |
| 0.4 | 154 | 2 | -40.2 | 1.2 |
| 0.6 | 1236 | 12 | -42.9 | 0.8 |
| 0.8 | 1711 | 25 | 24 | 0.8 |
| 1 | 1765 | 32 | 22.6 | 0.6 |
| 1.5 | 250 | 1 | 26.5 | 1 |
| 3 | 173 | 1 | 30.8 | 1.9 |
| 7 | 124.2 | 0,6 | 28.9 | 1.6 |

**Table S3:** Data shown in Figure 3A-3D.

| Figure 3A | | | | |
|---|---|---|---|---|
| **N/P ratio** | **Viability** (%) | **SD viability** (%) | **Viable cells** ($\cdot 10^6$ cells/mL) | **SD viable cells** ($\cdot 10^6$ cells/mL) |
| 0.2 | 95.8 | 0.6 | 4.9 | 0.5 |
| 0.4 | 96.6 | 0.2 | 5.2 | 0.1 |
| 0.6 | 96.4 | 0.2 | 5.3 | 0.5 |
| 0.8 | 95.7 | 0.2 | 5.0 | 0.2 |
| 1 | 95.9 | 0.6 | 4.8 | 0.2 |
| 1.5 | 94.5 | 0.2 | 5.2 | 0.3 |
| 3 | 70 | 3 | 2.6 | 0.9 |
| 7 | 63.3 | 0.5 | 0.8 | 0.7 |
| Positive control | 97.6 | 0.4 | 4.17 | 0.03 |
| Negative control | 97.5 | 0.4 | 4.31 | 0.05 |

| Figure 3B | | | | |
|---|---|---|---|---|
| **N/P ratio** | **Viability** (%) | **SD viability** (%) | **Viable cells** ($\cdot 10^6$ cells/mL) | **SD viable cells** ($\cdot 10^6$ cells/mL) |
| 0.2 | 96.9 | 0.7 | 4.3 | 0.4 |
| 0.4 | 96.5 | 0.4 | 4.0 | 0.7 |
| 0.6 | 97.05 | 0.07 | 4.7 | 0.7 |
| 0.8 | 96.2 | 1.1 | 4.0 | 0.5 |
| 1 | 96.5 | 0.2 | 4.6 | 0.4 |
| 1.5 | 97.05 | 0.07 | 4.9 | 0.6 |
| 3 | 95.5 | 0.6 | 3.5 | 0.1 |
| 7 | 94.8 | 0.4 | 3.4 | 1.7 |
| Positive control | 97.6 | 0.4 | 4.2 | 0.3 |
| Negative control | 97.5 | 0.4 | 4.3 | 0.5 |

| Figure 3C | | | | |
|---|---|---|---|---|
| **N/P ratio** | **MIF** (arb. units) | **SD MFI** (arb. units) | **GFP+ cells** (%) | **SD GFP+ cells** (%) |
| 0.2 | 578 | 530 | 1.2 | 0.3 |
| 0.4 | 294 | 49 | 1.20 | 0.14 |
| 0.6 | 1636 | 117 | 8.9 | 0.3 |
| 0.8 | 3094 | 648 | 25 | 6 |
| 1 | 5452 | 764 | 18.4 | 1.8 |
| 1.5 | 8285 | 785 | 31.0 | 0.4 |
| 3 | 8125 | 738 | 31.5 | 1.1 |
| 7 | 2330 | 1696 | 6.9 | 0.6 |
| Positive control | 5433 | 54 | 84.6 | 1.6 |
| Negative control | 145 | 2 | 1.1 | 0.3 |

| **Figure 3D** | | | | |
|---|---|---|---|---|
| **N/P ratio** | **MIF** (arb. units) | **SD MFI** (arb. units) | **GFP+ cells** (%) | **SD GFP+ cells** (%) |
| 0.2 | 390 | 187 | 0.2 | 0.1 |
| 0.4 | 719 | 69 | 1.2 | 0.2 |
| 0.6 | 1267 | 444 | 6.3 | 0.2 |
| 0.8 | 1132 | 99 | 16 | 2 |
| 1 | 1222 | 104 | 13 | 2 |
| 1.5 | 886 | 611 | 2.2 | 1.1 |
| 3 | 180 | 45 | 0.2 | 0.1 |
| 7 | 138 | 37 | 0.05 | 0.07 |
| Positive control | 5433 | 54 | 84.6 | 1.6 |
| Negative control | 145 | 2 | 1.1 | 0.3 |

**Table S4:** $2^4$ full factorial design, responses and ANOVA statistical analysis. Cell transfection efficiency (% of GFP-positive cells) was measured by flow cytometry, and cell growth ($10^6$ cell/mL) and cell viability (% live cells) using a nucleocounter. A pseudo-quadratic term (curvature) was included to account for areas in the workspace with higher values than workspace limit values.

| Factors | -1 | 0 | 1 |
|---|---|---|---|
| NaCl concentration (mM) | 0 | 100 | 200 |
| Incubation time (min) | 0 | 10 | 20 |
| N/P Ratio | 0.5 | 1.5 | 2.5 |
| DNA concentration (μg/mL) | 0.5 | 1 | 1.5 |

| Experimental run | Independent variables | | | | Responses | | |
|---|---|---|---|---|---|---|---|
| | NaCl concentration | Incubation time | N/P Ratio | DNA concentration | Viability (%) | Cell growth ($10^6$ cells/mL) | Transfection efficiency (%) |
| 1 | -1 | -1 | -1 | -1 | 86 ± 2 | 1.68 ± 0.01 | 0.3 ± 0.2 |
| 2 | 1 | -1 | -1 | -1 | 87 ± 3 | 2.29 ± 0.08 | 0.4 ± 0.1 |
| 3 | -1 | 1 | -1 | -1 | 82 ± 3 | 1.38 ± 0.03 | 0.4 ± 0.1 |
| 4 | 1 | 1 | -1 | -1 | 83.3 ± 1.3 | 1.22 ± 0.16 | 0.4 ± 0.1 |
| 5 | -1 | -1 | 1 | -1 | 86.4 ± 0.8 | 2.2 ± 0.4 | 3.3 ± 0.1 |
| 6 | 1 | -1 | 1 | -1 | 89 ± 3 | 2.2 ± 0.3 | 11.9 ± 0.8 |
| 7 | -1 | 1 | 1 | -1 | 83.8 ± 0.9 | 1.84 ± 0.07 | 1.4 ± 0.4 |
| 8 | 1 | 1 | 1 | -1 | 87 ± 4 | 1.80 ± 0.13 | 10.5 ± 0.2 |
| 9 | -1 | -1 | -1 | 1 | 90.9 ± 1.9 | 2.3 ± 0.6 | 2.5 ± 0.2 |
| 10 | 1 | -1 | -1 | 1 | 89.3 ± 1.1 | 2.7 ± 0.5 | 0.9 ± 0.1 |
| 11 | -1 | 1 | -1 | 1 | 84.4 ± 0.9 | 0.98 ± 0.03 | 1.5 ± 0.4 |
| 12 | 1 | 1 | -1 | 1 | 81.9 ± 1.7 | 0.7 ± 0.4 | 0.8 ± 0.1 |
| 13 | -1 | -1 | 1 | 1 | 49.1 ± 0.2 | 1.12 ± 0.07 | 5.1 ± 0.1 |
| 14 | 1 | -1 | 1 | 1 | 46 ± 3 | 0.84 ± 0.09 | 5.4 ± 0.6 |
| 15 | -1 | 1 | 1 | 1 | 54 ± 4 | 1.01 ± 0.19 | 5.2 ± 0.1 |

| | | | | | | | |
|---|---|---|---|---|---|---|---|
| 16 | 1 | 1 | 1 | 1 | 48 ± 3 | 0.80 ± 0.10 | 5.8 ± 0.5 |
| 17 | 0 | 0 | 0 | 0 | 93 ± 4 | 2.00 ± 0.02 | 17.1 ± 1.7 |
| 18 | 0 | 0 | 0 | 0 | 72 ± 33 | 1.7 ± 0.8 | 11 ± 9 |

| **Model** | F test. *p-value*[a] | Curvature test. *p-value* | $R^2$ |
|---|---|---|---|
| (A) Cell viability | **** | **** | 0.96 |
| (B) Cell growth | **** | * | 0.65 |
| (C) Transfection Efficiency | **** | **** | 0.88 |

| **Parameters Model (A)** | Coefficient | *F-value* |
|---|---|---|
| Constant | 76.6 | 246.6 |
| N/P Ratio | -8.8 | 226.9 |
| DNA concentration | -8.8 | 224.3 |
| N/P Ratio x DNA concentration | -10.0 | 288.7 |
| Curvature | 17.6 | 77.0 |

| **Parameters Model (B)** | Coefficient | *F-value* |
|---|---|---|
| Constant | 1.56 | 13.67 |
| Inc. Time | -0.35 | 24.49 |
| N/P Ratio | -0.09 | 1.61 |
| DNA concentration | -0.26 | 13.33 |
| N/P Ratio x DNA concentration | -0.28 | 15.24 |
| Curvature | 0.50 | 4.33 |

| **Parameters Model (C)** | Coefficient | *F-value* |
|---|---|---|
| Constant | 3.45 | 39.86 |
| NaCl concentration | 1.02 | 19.29 |
| N/P Ratio | 2.58 | 122.95 |
| DNA concentration | -0.08 | 0.14 |
| NaCl concentration x N/P Ratio | 1.29 | 30.74 |
| NaCl concentration x DNA concentration | -1.19 | 26.17 |
| Curvature | | 295.87 |

[a]*p-value* codification <: *: 0.05. **: 0.01. ***: 0.001****: 0.0001. Inc.Time: incubation time

**Table S5:** Ranges for NaCl concentration, N/P ratio, and pDNA concentration, experimental matrix, results and ANOVA analysis of the equations obtained in the CCD.

**Central Composite Design**

| Variables | Coding levels | | | | |
|---|---|---|---|---|---|
| | -α | -1 | 0 | 1 | α |
| N/P ratio | 0.3 | 0.8 | 1.5 | 2.2 | 2.7 |
| DNA concentration (µg/mL) | 0.2 | 0.5 | 1 | 1.5 | 1.84 |
| NaCl concentration (mM) | 16 | 50 | 100 | 150 | 184 |

| Experimental run | Independent variables | | | Responses | | | |
|---|---|---|---|---|---|---|---|
| | N/P Ratio | DNA concentration | NaCl concentration | Viability (%) | Cell growth (cells·$10^6$/mL) | Transfection efficiency (%) | GFP production (mg/L) |
| 1 | -1 | -1 | -1 | 98.9 | 4.57 | 0.9 | 0.008 |
| 2 | 1 | -1 | -1 | 97.8 | 5.02 | 1.95 | 0.012 |
| 3 | -1 | 1 | -1 | 92.6 | 3.93 | 25.85 | 0.054 |
| 4 | 1 | 1 | -1 | 95.4 | 4.05 | 6.2 | 0.016 |
| 5 | -1 | -1 | 1 | 98.25 | 5.49 | 3.7 | 0.013 |
| 6 | 1 | -1 | 1 | 97.6 | 5.30 | 1.8 | 0.03 |
| 7 | -1 | 1 | 1 | 93.85 | 4.15 | 22.25 | 0.038 |
| 8 | 1 | 1 | 1 | 59.1 | 3.81 | 32.9 | 0.117 |
| 9 | -α | 0 | 0 | 98.35 | 4.81 | 0.65 | 0.01 |
| 10 | α | 0 | 0 | 92.1 | 3.27 | 28.6 | 0.064 |
| 11 | 0 | -α | 0 | 95.95 | 7.30 | 0.45 | 0.019 |
| 12 | 0 | α | 0 | 93 | 3.97 | 28.1 | 0.118 |
| 13 | 0 | 0 | -α | 98.25 | 3.58 | 11.4 | 0.018 |
| 14 | 0 | 0 | α | 17.4 | 0.71 | 24.95 | 0.076 |
| 15 | 0 | 0 | 0 | 96.5 | 3.97 | 30.4 | 0.171 |
| 16 | 0 | 0 | 0 | 96.7 | 3.97 | 29.2 | 0.165 |
| 17 | 0 | 0 | 0 | 97 | 3.97 | 29.7 | 0.177 |
| 18 | 0 | 0 | 0 | 95.1 | 4.33 | 33.7 | 0.174 |

**Analysis**

| Model | F test. *p-value*[a] | Lack of fit test. *p-value* | $R^2$ | Adjusted $R^2$ | Predicted $R^2$ |
|---|---|---|---|---|---|
| (A) Cell viability | *** | < 0.0001 | 0.60 | 0.55 | -0.01 |
| (B) Cell growth | *** | 0.0038 | 0.74 | 0.67 | 0.15 |
| (C) Transfection efficiency | *** | 0.0007 | 0.81 | 0.72 | 0.35 |
| (D) GFP production | **** | 0.012 | 0.96 | 0.95 | 0.89 |

| **Parameters Model (A)** | Coefficient | *F-value* | *p-value* |
|---|---|---|---|

| | | | |
|---|---|---|---|
| Constant | 98.6 | 13.06 | *** |
| NaCl concentration | -12.6 | 13.32 | ** |
| (NaCl concentration)$^2$ | -11.9 | 12.79 | ** |
| **Parameters Model (B)** | Coefficient | *F-value* | *p-value* |
| Constant | 4.17 | 10.91 | *** |
| DNA concentration | -0.74 | 15.69 | ** |
| NaCl concentration | -0.27 | 2.06 | > 0.05 |
| (DNA concentration)$^2$ | 0.66 | 13.43 | ** |
| (NaCl concentration)$^2$ | -0.57 | 10.14 | ** |
| **Parameters Model (C)** | Coefficient | *F-value* | *p-value* |
| Constant | 30.92 | 9.16 | *** |
| N/P ratio | 2.72 | 2.04 | > 0.05 |
| DNA concentration | 9.18 | 23.26 | *** |
| NaCl concentration | 3.55 | 3.49 | > 0.05 |
| (N/P ratio)$^2$ | -6.32 | 11.66 | ** |
| (DNA concentration)$^2$ | -6.45 | 12.12 | ** |
| (NaCl concentration)$^2$ | -5.07 | 7.49 | * |
| **Parameters Model (D)** | Coefficient | *F-value* | *p-value* |
| Constant | 0.172 | 96.52 | **** |
| N/P ratio | 0.011 | 18.76 | ** |
| DNA concentration | 0.024 | 86.67 | **** |
| NaCl concentration | 0.015 | 33.93 | *** |
| N/P ratio x DNA concentration | 0.003 | 0.5485 | <0.05 |
| N/P ratio x NaCl concentration | 0.016 | 23.17 | ** |
| DNA concentration x NaCl concentration | 0.008 | 5.27 | * |
| (N/P ratio)$^2$ | -0.049 | 379.93 | **** |
| (DNA concentration)$^2$ | -0.038 | 226.92 | **** |
| (NaCl concentration)$^2$ | -0.046 | 327.11 | **** |
| N/P ratio x DNA concentration x NaCl concentration | 0.013 | 14.83 | ** |

[a]*p-value* codification <: *: 0.05. **: 0.01. ***: 0.001****: 0.0001
